# Statistical Model Checking for Variability-Intensive Systems

**DOI:** 10.1007/978-3-030-45234-6_15

**Published:** 2020-03-13

**Authors:** Maxime Cordy, Mike Papadakis, Axel Legay

**Affiliations:** 8 University of Paderborn, Paderborn, Germany; 9 ICREA, Open University of Catalonia, Barcelona, Spain; 10 SnT, University of Luxembourg, Luxembourg, Luxembourg; 11 Université Catholique de Louvain, Leuven, Belgium

## Abstract

We propose a new Statistical Model Checking (SMC) method to discover bugs in variability-intensive systems (VIS). The state-space of such systems is exponential in the number of variants, which makes the verification problem harder than for classical systems. To reduce verification time, we sample executions from a featured transition system – a model that represents jointly the state spaces of all variants. The combination of this compact representation and the inherent efficiency of SMC allows us to find bugs much faster (up to 16 times according to our experiments) than other methods. As any simulation-based approach, however, the risk of Type-1 error exists. We provide a lower bound and an upper bound for the number of simulations to perform to achieve the desired level of confidence. Our empirical study involving 59 properties over three case studies reveals that our method manages to discover all variants violating 41 of the properties. This indicates that SMC can act as a low-cost-high-reward method for verifying VIS.

### 1 Introduction

We consider the problem of bug detection in Variability Intensive Systems (VIS). This category of systems encompasses any system that can be derived into multiple variants (differing, e.g., in provided functionalities), including software product lines [12] and configurable systems [32]. Compared to traditional ("single") systems, the complexity of bug detection in VIS is increased: bugs can appear only in some variants, which requires analysing the peculiarities of each variant.

Among the number of techniques developed for bug detection, one finds testing and model checking. Testing [6] executes particular test inputs on the system and checks whether it triggers a bug. Albeit testing remains widely used in industry, the rise of concurrency and inherent system complexity has made system-level test case generation a hard problem. Also, testing is often limited to bounded reachability properties and cannot assess liveness properties.

Model checking [2] is a formal verification technique which checks that all behaviours of the system satisfy specified requirements. These behaviours are typically modelled as an automaton, whose each node represents a state of the system (e.g. a valuation of the variables of a program and a location in this program's execution flow) and where each transition between two states expresses that the program can move from one state to the other by executing a single action (e.g. executing the next program statement). Requirements are often expressed in temporal logics, e.g. the Linear Temporal Logic (LTL) [31].

Such logics capture both safety and liveness properties of system behaviours. As an example, consider the LTL formula $\Box(command\_sleep \Rightarrow \Diamond system\_sleep)$. *command_sleep* and *system_sleep* are logic atoms and represent, respectively, a state where the `sleep` command is input and another state where the system enters sleep mode. The symbols $\Box$ and $\Diamond$ means *always* and *eventually*, respectively. Thus, the whole formula expresses that "it is always the case that when the `sleep` command is input, the system eventually enters sleep mode".

Contrary to testing, model checking is exhaustive: if a bug exists then the checking algorithm outputs a *counterexample*, i.e. an execution trace of the system that violates the verified property. Exhaustiveness makes model checking an appealing solution to obtain strong guarantees that the system works as intended. It can also nicely complement testing (whose main advantage remains to be applied directly on the running system), e.g. by reasoning over liveness properties or by serving as oracle in test generation processes [1]. Those benefits, however, come at the cost of scalability issues, the most prominent being the *state explosion problem.* This term refers to the phenomenon where the state space to visit is so huge that an exhaustive search is intractable. As an illustration of this, let us remark that the theoretical complexity of the LTL model-checking problem is PSPACE-complete [37].

Model checking complexity is further exacerbated when it comes to VIS. Indeed, in this case, the model-checking problem requires verifying whether *all* the variants satisfy the requirements [11]. This means that, if the VIS comprises $n$ variation points ($n$ features in a software product line or $n$ Boolean options in a configurable system), the number of different variants to represent and to check can reach $2^n$. This exponential factor adds to the inherent complexity of model checking. Thus, checking each variant (or models thereof) separately – an approach known as *enumerative* or *product-based* [34] – is often intractable. To alleviate this, variability-aware models and companion algorithms were proposed to represent and check efficiently the behaviour of all variants at once. For instance, *Featured Transition Systems* (FTS) [11] are transition systems where transitions are labelled with (a symbolic encoding of) the set of variants able to exercise this transition. The structure of FTS, if well constructed, allows one to capture in a compact manner commonalities between states and transitions of several variants. Exploiting that information, *family-based* algorithms can check only once the executions that several variants can execute and explore the state space of an individual variant only when it differs from all the others. In spite of positive improvements over the enumerative approach, state-space explosion remains a major challenge.

In this work, we propose an alternative technique for state-space exploration and bug detection in VIS. We use Statistical Model Checking (SMC) [26] as a trade-off between testing and model checking to verify properties (expressed in full LTL) on FTS. The core idea of SMC is to conduct some simulations (i.e. sample executions) of the system (or its model) and verify if these executions satisfy the property to check. The results are then used together with statistical tests to decide whether the system satisfies the property with some degree of confidence. Of course, in contrast with an exhaustive approach, a simulation-based solution does not guarantee a result with 100% confidence. Still, it is possible to bound the probability of making an error. Simulation-based methods are known to be far less memory- and time-consuming than exhaustive ones, and are sometimes the only viable option. Over the past years, SMC has been used to, e.g. assess the absence of errors in various areas from aeronautic to systems biology; measure cost average and energy consumption for complex applications such as nanosatellites; detect rare bugs in concurrent systems [10, 21, 25].

Given an LTL formula and an FTS, our *family-based* SMC method samples executions from all variants at the same time. Doing so, it avoids sampling twice (or more) executions that exist in multiple variants. Merging the individual state spaces biases the results, though, as it changes the probability distribution of the executions. This makes the problem different from previous methods intended for single systems (e.g. [20]) and obliges us to revisit the fundamentals of SMC in the light of VIS. In particular, we want to characterize the number of execution samples required to bound the probability of Type-1 error by a desired degree of confidence. We provide a lower bound and an upper bound for this number by reducing its computation to particular instances of the coupon problem [4]. We implemented our method within ProVeLines [17], a model checker for VIS. We provide empirical evidence, based on 3 case studies totalling 59 properties to check, that family-based SMC is a viable approach to verify VIS. Our study shows that our method manages to find all buggy variants in 41 properties and does so up to 16 times faster than state-of-the-art model-checking algorithms for VIS [11]. Moreover, our approach can achieve a median bug detection rate 3 times higher than classical SMC applied to each variant individually. The hardest cases arise when the state space of some variant is substantially smaller than the other. This leads to a reduced probability to find a bug in those variants.

### 2 Background on Model Checking

In model checking, the behaviour of the system is often represented as a transition system $(S, \Delta, AP, L)$ where $S$ is a set of states, $\Delta \subseteq S \times S$ is the transition relation, $AP$ is a set of atomic propositions[3] and $L : S \to 2^{AP}$ labels any state with the atomic propositions that the system satisfies when in such a state.

#### 2.1 Linear Temporal Logic

LTL is a temporal logic that allows specifying desired properties over all future executions of some given system. Given a set $AP$ of atomic propositions, an LTL

[3] Atomic propositions can be seen as basic observable properties of the system state.

formula $\phi$ is formed according to the following grammar: $\phi ::= \top \mid a \mid \phi_1 \wedge \phi_2 \mid \neg\phi_1 \mid \bigcirc\phi_1 \mid \phi_1 \mathrm{U} \phi_2$ where $\phi_1$ and $\phi_2$ are LTL formulae, $a \in AP$, $\bigcirc$ is the next operator and U is the until operator. We also define $\Diamond\phi$ ( *"eventually"* $\phi$) and $\Box\phi$ ( *"always"* $\phi$) as a shortcut for $\top\mathrm{U}\phi$ and $\neg\Diamond\neg\phi$, respectively.

Vardi and Wolper have presented an automata-based approach for checking that a system – modelled as a transition system $ts$ – satisfies an LTL formula $\phi$ [37]. Their approach consists of, first, transforming $\phi$ into a Büchi automaton $\mathcal{B}_{\neg\phi}$ whose language is exactly the set of executions that violate $\phi$, that is, those that visit infinitely often a so-called *accepting* state. Such execution $\sigma$ takes the form of a *lasso*, i.e. $\sigma = q_0 \ldots q_n$ with $q_j = q_n$ for some $j$ and where $q_i$ is accepting for some $i : j \leq i \leq n$. We name *accepting* any such lasso whose cycle contains an accepting state.

The second step is to compute the synchronous product of $ts$ and $\mathcal{B}_{\neg\phi}$, which results in another Büchi automaton $\mathcal{B}_{ts\otimes\neg\phi}$. Any accepting lasso in $\mathcal{B}_{ts\otimes\neg\phi}$ represents an execution of the system that violates $\phi$. Thus, Vardi and Wolper's algorithm comes down to checking the absence of such accepting lasso in the whole state space of $\mathcal{B}_{ts\otimes\neg\phi}$. The size of this state space is $\mathcal{O}(|ts| \times |2^{|\phi|}|)$ and the complexity of this algorithm is PSPACE-complete.

#### 2.2 Statistical Model Checking

Originally, SMC was used to compute the probability to satisfy a bounded LTL property for stochastic system [39]. The idea was to monitor the properties on bounded executions represented by Bernoulli variables and then use Monte Carlo to estimate the resulting property. SMC also applies to non-stochastic systems by assuming an implicit uniform probability distribution on each state successor.

Grosu and Smolka [20] lean on this and propose an SMC method to address the full LTL model-checking problem. Their sampling algorithm walks randomly through the state space of $\mathcal{B}_{ts\otimes\neg\phi}$ until it finds a lasso. They repeat the process $M$ times and conclude that the system satisfies the property if and only if none of the $M$ lassos is accepting. They also show that, given a confidence ratio $\delta$ and assuming that the probability $p$ for an execution of the system exceeds an error margin $\epsilon$, setting $M = \frac{\delta}{1-\epsilon}$ bounds the probability of a Type-1 error (rejecting the hypothesis that the system violates the property while it actually violates it) by $\delta$. Thus, $M$ can serve as a minimal number of samples to perform. Our work extends theirs in order to support VIS instead of single systems. Other work on applying SMC to the full LTL logic can be found in [18,38].

#### 2.3 Model Checking for VIS

Applying classical model checking to VIS requires iterating over all variants, construct their corresponding automata $\mathcal{B}_{ts\otimes\neg\phi}$ and search for accepting lasso in each of these. This enumerative method (also named *product-based* [34]) fails to exploit the fact that variants have behaviour in common.

As an alternative, researchers came up with models able to capture the behaviour of multiple variants and distinguish between the unique and common

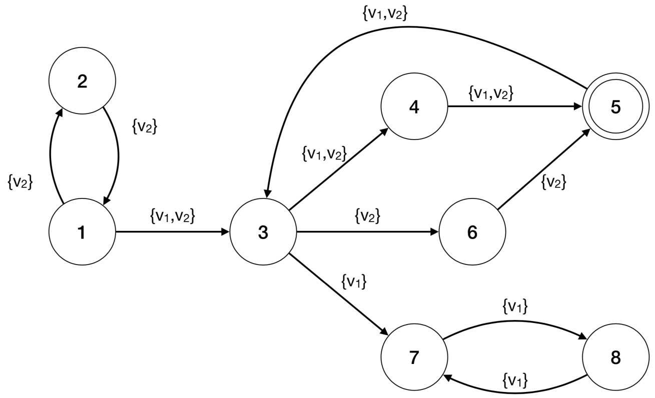

Fig. 1: An example of FBA with two variants.

behaviour of those variants [3, 8, 11]. Among such models, we focus on *featured transition systems* [11] as those can link an execution to the variants able to execute it more directly than the alternative formalisms. In a nutshell, FTS extend the standard transition system by labelling each transition with a symbolic encoding of the set of variants able to exercise this transition. Then, the set of variants that can produce an execution $\pi$ is the intersection of all sets of variants associated with the transitions in $\pi$.

To check which variants violate a given LTL formula $\phi$, one can adapt the procedure of Vardi and Wolper and build the synchronous product of the featured transition system with $\mathcal{B}_{\neg\phi}$ [11]. This product is similar to the Büchi automaton obtained in the single system case, except that its transitions are also labelled with a set of variants.[4] Then, the buggy variants are those that are able to execute the accepting lassos of this automaton. This generalized automaton is the fundamental formalism we work on in this paper.

**Definition 1.** Let $V$ be a set of variants. A *Featured Büchi Automaton* (FBA) over $V$ is a tuple $(Q,\, \Delta,\, Q_0,\, A,,\, \Theta,\, \gamma)$ where $Q$ is a set of states, $\Delta \subseteq Q \times Q$ is the transition relation, $Q_0 \subseteq Q$ is a set of initial states, $A \subseteq Q$ is the set of accepting states, $\Theta$ is the whole set of variants, and $\gamma : \Delta \to 2^{\Theta}$ associates each transition with the set of variants that can execute it.

Figure 1 shows an FBA with two variants and eight states. State 5 as the only accepting state. Both variants can execute the transition from State 3 to State 4, whereas only variant $v_2$ can move from State 3 to State 6.

The Büchi automaton corresponding to one particular variant $v$ is derived by removing the transitions not executable by $v$. That is, we remove all transitions $(q, q') \in \Delta$ such that $v \notin \gamma(q, q')$. The resulting automaton is named the *projection* of the FBA onto $v$. For example, one obtains the projection of the FBA in

[4] Those labels are equal to those found in the corresponding transitions of the featured transition system.

Figure 1 onto $v_2$ by removing the transition from State 3 to State 7 and those between State 7 to State 8.

#### 2.4 Other Related Work

Recent work has applied SMC in the context of VIS. In [36], the authors proposed an algebraic language to describe (quantitative) behavioural variability in a dynamic manner. While their work shares some similarities with ours, there are fundamental differences. First, we seek for guaranteeing the absence of bugs in all variants of the family (applying family-based concepts), while they focus on dynamic feature interactions (on a product-based basis). The second difference is that they consider quantitative bounded properties, while we support the entire LTL verification problem by extending the multi-lasso concept of [20,28].

Another related, yet different area is the sampling of VIS variants (e.g. [27, 30]). Such work considers the problem of sampling uniformly variants in order to study their characteristics (e.g. performance [22] and other quality requirements [15]) and infers those of the other variants. Recently, Thüm et al. [35] survey different strategies for the performance analysis of VIS, including the sampling of variants and family-based test generation, which is based on the same idea of executing test cases common to multiple variants. Contrary to us, such works do not consider temporal/behavioural properties and most of them perform the sampling based on a static representation of the variant space (i.e. a feature model [23]). An interesting direction for future work is to combine our family-based SMC with sampling techniques to check only representative variants of the family.

### 3 Family-Based Statistical Model Checking

The purpose of SMC is to reduce the verification effort (when visiting the state space of the system model) by sampling a given number of executions (i.e. lassos). This gain in efficiency, however, comes at the risk of Type-1 errors. Indeed, while the discovery of a counterexample leads with certainty to the conclusion that the variants able to execute it violate the property $\phi$, the fact that the sampling did not find a counterexample for some variant $v$ does not entail a 100% guarantee that $v$ satisfies $\phi$. The more lassos we sample, the more confident we can get that the variants without counterexamples satisfy $\phi$. Thus, designing a family-based SMC method involves answering three questions: (1) how to sample executions; (2) how to choose a suitable number of executions; (3) what is the associated probability of Type-1 error.

#### 3.1 Random Sampling in Featured Büchi Automata

One can sample a lasso in an FBA by randomly walking through its state space, starting from a randomly-chosen initial state and ending as soon as a cycle is found. A particular restriction is that this lasso should be executable by at least

**Input:** $fba = (Q,\, \Delta,\, Q_0,\, A,\, \Theta,\, \gamma)$
**Output:** $(\sigma,\, \Theta_\sigma, accept)$ where $\sigma$ is a lasso of $fba$ and $\Theta_\sigma$ is the set of the variants able to execute $\sigma$ and *accept* is true iff $\sigma$ is accepting.

1 $q_0 \leftarrow$ pick from $Q_0$ with probability $\frac{1}{|Q_0|}$;
2 $q \leftarrow q_0$; $\sigma \leftarrow q_0$; $\Theta_\sigma \leftarrow \Theta$; $depth \leftarrow 0$; $a \leftarrow 0$;
3 **while** $hash(q) = \bot$ **do**
4     $depth \leftarrow depth + 1$;
5     $hash(q) \leftarrow depth$;
6     **if** $q \in A$ **then**
7         $a \leftarrow depth$;
8     **end**
9     $Succ_\sigma \leftarrow \{q' \in Q | (q, q') \in \Delta \wedge (\gamma(q, q') \cap \Theta_\sigma) \neq \emptyset\}$;
10     $q' \leftarrow$ pick from $Succ_\sigma$ with probability $\frac{1}{|Succ_\sigma|}$;
11     $\sigma \leftarrow \sigma q'$;
12     $\Theta_\sigma \leftarrow \Theta_\sigma \cap \gamma(q, q')$;
13     $q \leftarrow q'$;
14 **end**
15 **return** $(\sigma, \Theta_\sigma, hash(q) \leq a)$

**Algorithm 1:** Random Lasso Sampling one variant; otherwise, we would sample a behaviour that does not actually exist. The set of variants able to execute a given lasso are those that can execute all its transitions, i.e. the intersection of all $\gamma(q, q')$ met along the transitions of this lasso. More generally, we define the lasso sample space of an FBA as follows.

**Definition 2.** Let $fba = (Q,\, \Delta,\, Q_0,\, A,\, \Theta,\, \gamma)$ be a featured Büchi automaton. The lasso sample space $L$ of $fba$ is the set of executions $\sigma = q_0 \ldots q_n$ such that $q_0 \in Q_0$, $(q_i, q_{i+1}) \in \Delta$ for all $0 \leq i \leq n-1$, $(\bigcap_{0 \leq i \leq n-1} \gamma(q_i, q_{i+1})) \neq \emptyset$, $q_j = q_n$ for some $0 \leq j \leq n-1$ and $a \neq b \Rightarrow q_a \neq q_b$ for all $0 \leq a, b \leq n-1$. Moreover, $\sigma$ is said to be an accepting lasso if $\exists q_a \in A$ for some $j \leq a \leq n$.

Algorithm 1 formalizes the sampling of lassos in a deadlock-free FBA.[5] After randomly picking an initial state (Line 1), we walk through the state space by randomly choosing, at each iteration, a successor state among those available (Line 7–18). Throughout the search, we maintain the set of variants $\Theta_\sigma$ that can execute $\sigma$ so far (Line 16). Then, we use this set as a filter when selecting successor states, so as to make sure that $\sigma$ remains executable by at least one variant. At Line 13, $Succ_\sigma$ is the set of successors $q'$ of $q$ (last state of $\sigma$) that can be reached. We stop the search as soon as we reach a state that was previously visited (Line 7). If this state was visited before the last accepting state, it means that the sampled lasso is accepting (Line 19).

[5] We assume that no variant may remain stuck in a state without outgoing transition that this variant can execute. Should this happen, we assume that the variant self-loops in the state wherein it is stuck, yielding an immediate lasso.

A motivated criticism [28] of the use of random walk to sample lasso is that shorter lassos receive a higher probability to be sampled. To counterbalance this, we implemented a heuristic named *multi-lasso* [20]. It consists of ignoring backward transitions that do not lead to an accepting lasso if there are still forward transitions to explore. This is achieved by modifying Line 13 such that backward transitions leading to a non-accepting lasso are not considered in the successor set.

Assuming a uniform selection of outgoing transitions from each state, one can compute the probability that a random walk samples any given lasso from the sample space.

**Definition 3.** The probability $P(\sigma)$ of a lasso $\sigma = q_0 \dots q_n$ is inductively defined as follows: $P[q_0] = |Q_0|^{-1}$ and $P[q_0 \dots q_j] = P[q_0 \dots q_{j-1}] \times |Succ_{q_0 \dots q_{j-1}}|^{-1}$.

In the absence of deadlock, $(L, \mathcal{P}(\mathcal{L}), P)$ defines a probability space. Probability spaces on infinite executions are by no means a trivial construction (see e.g. [9]). Nevertheless, the proof of this proposition is similar to its counterpart in Büchi automata [20] and is therefore omitted. It derives from the observation that the lasso sample space is composed of non-subsuming finite prefixes of all infinite paths of the automaton.

Let us consider an example. In the FBA from Figure 1, there are two non-accepting lassos ($l_1 = (1, 2, 1)$ and $l_2 = (1, 3, 7, 8, 7)$) and two accepting lassos ($l_3 = (1, 3, 4, 5, 3)$ and $l_4 = (1, 3, 6, 5, 3)$). Both variants can execute lassos $l_3$, while only $v_1$ can execute $l_2$ and only $v_2$ can execute $l_1$ and $l_4$. The probability of sampling $l_1$ is $\frac{1}{2}$, whereas $P[l_2] = P[l_3] = P[l_4] = \frac{1}{6}$. Thus, the probability of sampling a counterexample executable by $v_2$ is $\frac{1}{3}$, whereas it is only $\frac{1}{6}$ for $v_1$.

Next, we characterize the relationship between this probability space and any individual variant $v$. Let $L_v$ be the set of lassos executable by $v$. Since $L_v \subseteq L$, the probability $p_v$ to sample such a lasso is $\sum_{\sigma_v \in L_v} P(\sigma)$. Note that $p_v$ can be different from the probability $\hat{p}_v$ of sampling an accepting lasso from the automaton modelling the behaviour of $v$ only (i.e. the projection of the FBA onto $v$). This is because, in the FBA, the probability of selecting an outgoing transition from a given state is assigned uniformly regardless of the number of variants able to execute that transition. This balance-breaking effect increases more as the variants have different numbers of unique executions.

Let $\sigma = q_0 \dots q_n$ be a lasso in $L_v$. Then $P_v(\sigma)$ is inductively defined as follows: $P_v[q_0] = P[q_0]$ and $P_v[q_0 \dots q_j] = P_v[q_0 \dots q_{j-1}] \times |\{(q_{j-1}, q) \in \Delta_v : q \in Q\}|^{-1}$ where $\Delta_v = \{(q, q') \in \Delta : v \in \gamma(q, q')\}$. In our example, $P_{v_1}[l_3] = \frac{1}{2}$, as opposed to $P[l_3] = \frac{1}{6}$. This implies that it is more likely to sample an accepting lasso executable by $v_1$ from its projection in one trial than it is from the whole FBA in two trials. This illustrates the case where merging the state spaces of the variants can have a negative impact on the capability to find bugs specific to one variant.

Thus, sampling lassos from the FBA allows finding one counterexample executable by multiple products but it introduces a bias. Overall, it tends to decrease the probability of sampling lassos from variants that have a smaller state space.

This can impact the results and parameter choices of SMC, like the number of samples required to get confident results and the associated Type-1 error.

#### 3.2 Hypothesis Testing

Remember that addressing the model checking problem for VIS requires to find a counterexample for every buggy variant $v$. Thus, one must sample a number $M$ of lassos such that one gets an accepting lasso for each such buggy variant with a confidence ratio $\delta$. Let $fba$ be a featured Büchi automaton, $v$ be a variant and $p_v = \sum \sigma \in L_v^\omega P(\sigma)$ where $L_v^\omega$ is the set of accepting lasso executable by $v$. Let $Z_v$ denote a Bernoulli random variable such that $Z_v = 1$ with probability $p_v$ and $Z_v = 0$ with probability $q_v = 1 - p_v$. Now, let $X_v$ denote the geometric random variable with parameter $p_v$ that encodes the number of independent samples required until $Z_v = 1$. For a set of variants $V = \{v_1 \dots v_{|V|}\}$, we have that $X_{v_1} \dots X_{v_{|V|}}$ are *not* independent since one may sample a lasso executable by more than one variant.

We define $X = \max_{i=1..|V|} X_{v_i}$. We aim to find a number of sample $M$ such that $P[X \leq M] \geq 1 - \delta$ for a confidence ratio $\delta$. This is analogous to the coupon collector's problem [4], which asks how many boxes are needed to collect one instance of every coupon placed randomly in the boxes. It differs from the standard formulation in that the probability of occurrence of coupons are neither independent nor uniform, and a single box can contain 0 to $|V|$ coupons. Even for simpler instances of the coupon problem, computing $P[X \leq M]$ analytically is known to be hard [33]. Thus, existing solutions rather characterise a lower bound and an upper bound. We follow this approach as well.

#### 3.3 Lower Bound (Minimum Number of Samples)

To compute a lower bound for the number of samples to draw, we transform the family-based SMC problem to a simpler form (in terms of verification effort). We divide our developments into two parts. First, we show that assigning equal probabilities $p_{v_i}$ to every variant $v_i$ (obtained by averaging the original probability values) reduces the number $M$ of required samples. As a second step, we show that assuming that all variants share all their executions also reduces $M$. Doing so, we reduce the family-based SMC problem to its single-system counterpart, which allows us to obtain the desired lower bound.

**Averaged probabilities.** Let $p_{avg} = \frac{1}{|V|} \sum_{v=1..|V|} p_v$ and $X_{\mathbf{even}}$ be the counterpart of $X$ where all probabilities $p_{v_i}$ have been replaced by $p_{avg}$.

**Lemma 4.** *For any number $N$, it holds that $P[X_{\boldsymbol{even}} \leq N] \geq P[X \leq N]$.*

Intuitively, the value of $X$ depends mainly on the variants whose accepting lassos are rarer. By averaging the probability of sampling accepting lassos, we raise the likelihood to get those rarer lassos and, thus, the number of samples required to get an accepting lasso for all variants. Shioda [33] proves a similar result for the coupon collector problem. He does so by showing that the vector $\mathbf{p_{even}}$ *majorizes* $\mathbf{p} = \{p_{v_1} \dots p_{v_1}\}$ and that the *ccdf*[6] of $X$ is a Schur-concave function of the sampling probabilities. Even though our case is more general than the non-uniform coupon collector's problem, the result of Lemma 4 still holds. Indeed, we observe that the theoretical proof of [33] (a) does not assume the independence of the random variables $Z_{v_i}$; (b) still applies to the dependent case; and (c) supports the case where the sum of the probability values $p_{v_i}$ is less than one.

**Maximized commonalities.** Next, let $X_{\mathbf{all}}$ be the particular case of $X_{\mathbf{even}}$ where all accepting lassos are executable by all variants and are sampled with probability $p_{avg}$. Thus, the number of samples to find an accepting lasso for every variant is reduced to the number of samples required to find any accepting lasso.

**Lemma 5.** *It holds that* $P[X_{\mathbf{all}} \leq N] \geq P[X_{\mathbf{even}} \leq N]$.

Moreover, let us note that $X_{\mathbf{all}}$ is a geometric random variable with parameter $p_{avg}$. This reduces our problem to sampling an accepting lasso in a classical Büchi automaton and allows us to reuse the results of Grosu and Smolka [20].

**Lemma 6.** *For a confidence ratio* $\delta$ *and an error margin* $\epsilon$*, it holds that*

$$p_{avg} \geq \epsilon \Rightarrow P[X_{\mathbf{all}} \leq M] \geq P[X_{\mathbf{all}} \leq N] = 1 - \delta$$

*where* $M = \frac{ln(\delta)}{ln(1-\epsilon)}$ *and* $N = \frac{ln(\delta)}{ln(1-p_{avg})}$.

This leads us to the central result of this section.

**Theorem 7.** *Assuming that* $p_{avg} \geq \epsilon_{avg}$ *for a given error margin* $\epsilon_{avg}$*, a lower bound for the number of samples required to find an accepting lasso for each buggy variant is* $M = \frac{ln(\delta)}{ln(1-\epsilon_{avg})}$ *with a Type-1 error bounded by* $\delta$.

#### 3.4 Upper Bound (Maximum Number of Samples)

We follow a similar two-step process to characterise an upper bound for $M$. In the first step, we replace the probabilities $p_{v_i}$ of every variant by their minimum. In the second step, we alter the model so that the variants have no common behaviour. Then we show that, given a desired degree of confidence, the obtained model requires a higher number of samples than the original one.

**Minimum probability.** Let $p_{min} = \min_{v=1..|V|} p_v$ and $X_{\mathbf{min}}$ be the counterpart of $X$ where all probabilities $p_{v_i}$ have been replaced by $p_{min}$. The *ccdf* of $X$ being a decreasing function of the sampling probabilities, we have that $P[X_{\mathbf{min}} \leq N] \leq P[X \leq N]$.

[6] *ccdf* stands for complementary cumulative distribution function

**No common counterexamples.** Let $\{(X_{\mathbf{indep}})_{v_i}\}$ be a set of independent geometric random variables with parameters $p_{min}$ and let $X_{\mathbf{indep}} = \max(X_{\mathbf{indep}})_{v_i}$. $X_{\mathbf{indep}}$ actually encodes the number of samples required to get a counterexample for all buggy variants when those have no common counterexamples. We have that $P[X_{\mathbf{indep}} \leq N] \leq P[X_{\mathbf{min}} \leq N]$, since the number of samples to perform cannot be reduced by sampling a counterexample executable by multiple variants. Now, let us note that $X_{\mathbf{indep}}$ is an instance of the uniform coupon problem with $|V|$ coupons to collect. A lower bound for $P[X_{\mathbf{indep}} \leq M]$ is known to be $1 - |V| \times (1 - p_{min})^M$ [33]. Assuming $p_{min}$ greater than some error margin $\epsilon_{min}$, we have $P[X_{\mathbf{indep}} \leq M] \geq 1 - |V| \times (1 - \epsilon_{min})^M$. Setting a confidence ratio $\delta$, we want to find a $M$ such that $P[X_{\mathbf{indep}} \leq M] \geq 1 - \delta$. By solving $1 - |V|(1 - \epsilon_{min})^M = 1 - \delta$, we obtain $M = \frac{ln(\delta) - ln(|V|)}{ln(1-\epsilon_{min})}$, which we can use as the upper bound for the number of samples to perform.

**Theorem 8.** *Assuming that $p_{min} \geq \epsilon_{min}$ for a given error margin $\epsilon_{min}$, an upper bound for the number of samples required to find an accepting lasso for each buggy variant is $M = \frac{ln(\delta) - ln(|V|)}{ln(1-\epsilon_{min})}$ with a Type-1 error is bounded by $\delta$.*

### 4 Empirical Study

#### 4.1 Objectives and Methodology

One can regard SMC as a means of speeding up verification while risking missing counterexamples. Our first question studies this trade-off and analyses the empirical Type-1 error rate. More precisely, we compute the detection rate of our family-based SMC method, expressed as the number of buggy variants that it detects over the total number of buggy variants.

**RQ1** *What is the empirical buggy variant detection rate of family-based SMC?*

We compute the detection rate for different numbers $M$ of samples lying between the lower and upper bounds as characterised in Section 3. To get the ground truth (i.e. the true set of all buggy variants), we execute the exhaustive LTL model checking algorithms for FTS designed by Classen et al. [11]. For the lower bound, we assume that the average probability to sample an accepting lasso for any variant is higher than $\epsilon_{avg} = 0.01$. Setting a confidence ratio $\delta = 0.05$ yields $\frac{ln(0.05)}{ln(0.99)} = 298$. We round up and set $M = 300$ as our lower bound. For the higher bound, we assume that the minimum probability to sample a counterexample in a buggy variant is higher than $\epsilon_{min} = 3.10^{-4}$ and also set $\delta = 0.05$. For a model with 256 variants[7], this yields $M = \frac{ln(0.05) - ln(256)}{ln(0.9997)} = 18478$. For convenience, we round it up to $19,200 = 300 \cdot 2^6$. In the end, we successively set $M$ to $300, 600, \ldots, 19200$ and observe the detection rates.

Next, we investigate a complementary scenario where the engineer has a limited budget of samples to check. We study the smallest budget required by

[7] 256 is the maximum number of variants in our case studies

SMC to detect all buggy variants (in the cases where it can indeed detect all of them) and what is the incurred computation resources compared to an exhaustive search of the state space. Thus, our second question is:

**RQ2** *How much efficient is SMC with a minimal sample budget compared to an exhaustive search?*

Finally, we compare family-based SMC with the alternative of sampling in each variant's state space separately. We name this alternative method *enumerative SMC*. Hence, our last research question is:

**RQ3** *How does family-based SMC compares with enumerative SMC?*

As before, we compare the two techniques w.r.t. detection rate. We set $M$ to the same values as in RQ1. In enumerative SMC, this means that each variant receives a budget of samples of $\frac{M}{|V|}$ where $M$ is the number of samples used in family-based SMC and $V$ is the set of variants.

#### 4.2 Experimental Setup

*Implementation.* We implemented our SMC algorithms (family-based and enumerative-based) in a prototype tool. The tool takes as input an FTS, an LTL formula and a sample budget. Then it performs SMC until all samples are checked or until all variants are found to violate the formula. To compare with the exhaustive search we use ProVeLines [17], a state-of-the-art model checker for VIS.

*Dataset.* We consider three systems that were used in past research to evaluate VIS model checking algorithms [11,14,16]. Table 1 summarizes the characteristics of our case studies and their related properties. The first system is a minepump system [11,24] with 128 variants. The underlying FTS comprises 250,561 states, while the state space of all variants taken individually reaches 889,124 states. The second model is an elevator model inspired by Plath and Ryan [29]. It is composed of eight configuration options, which can be combined into 256 different variants, and its FTS has 58,945,690 states to explore. The third and last is a case study inspired by the CCSDS File Delivery Protocol (CFDP) [13], a real-world configurable spacecraft communication protocol [5]. The FTS modelling the protocol consists of 1,732,536 states to explore and 56 variants (individually totalling 2,890,399 states). We discarded the properties that are satisfied by all variants. Those are: Minepump #17, #33, #40; Elevator #13, CFDP #5. Indeed, these properties are not relevant for RQ1 and RQ3 since SMC is trivially correct in such cases. As for RQ2, any small sample budget would return correct results while being more efficient than the exhaustive search. This leaves us with 59 properties.

*Infrastructure and repetitions.* We run our experiments on a MacBook Pro 2018 with a 2.9 GHz Core-i7 processor and macOS 10.14.5. To account for random variations in the sampling, we execute 100 runs of each experiment and compute the average detection rates for each property.

Table 1: Models and LTL formulae used in our experiments.

| | |
|---|---|
| **Minepump** (250,561 FTS states, 128 valid variants) | |
| #1 | $\neg(\Box\Diamond(stateReady \wedge highWater \wedge userStart))$ |
| #2 | $\neg(\Box\Diamond stateReady)$ |
| #3 | $\neg(\Box\Diamond stateRunning)$ |
| #4 | $\neg(\Box\Diamond stateStopped)$ |
| #5 | $\neg(\Box\Diamond stateMethanestop)$ |
| #6 | $\neg(\Box\Diamond stateLowstop)$ |
| #7 | $\neg(\Box\Diamond readCommand)$ |
| #8 | $\neg(\Box\Diamond readAlarm)$ |
| #9 | $\neg(\Box\Diamond readLevel)$ |
| #10 | $\neg((\Box\Diamond readCommand) \wedge (\Box\Diamond readAlarm) \wedge (\Box\Diamond readLevel))$ |
| #11 | $\neg(\Box\Diamond pumpOn)$ |
| #12 | $\neg(\Box\Diamond\neg pumpOn)$ |
| #13 | $\neg((\Box\Diamond pumpOn) \wedge (\Box\Diamond\neg pumpOn))$ |
| #14 | $\neg(\Box\Diamond methane)$ |
| #15 | $\neg(\Box\Diamond\neg methane)$ |
| #16 | $\neg((\Box\Diamond methane) \wedge (\Box\Diamond\neg methane))$ |
| #17 | $\Box(\neg pumpOn \vee stateRunning)$ |
| #18 | $\Box(methane \Rightarrow (\Diamond stateMethanestop))$ |
| #19 | $\Box(methane \Rightarrow \neg(\Diamond stateMethanestop))$ |
| #20 | $\Box(pumpOn \vee \neg methane)$ |
| #21 | $\Box((pumpOn \wedge methane) \Rightarrow \Diamond\neg pumpOn)$ |
| #22 | $((\Box\Diamond readCommand) \wedge (\Box\Diamond readAlarm) \wedge (\Box\Diamond readLevel)) \Rightarrow \Box((pumpOn \wedge methane) \Rightarrow \Diamond\neg pumpOn)$ |
| #23 | $\neg\Diamond\Box(pumpOn \wedge methane)$ |
| #24 | $((\Box\Diamond readCommand) \wedge (\Box\Diamond readAlarm) \wedge (\Box\Diamond readLevel)) \Rightarrow \neg\Diamond\Box(pumpOn \wedge methane)$ |
| #25 | $\Box((\neg pumpOn \wedge methane \wedge \Diamond\neg methane) \Rightarrow ((\neg pumpOn) U \neg methane))$ |
| #26 | $\Box((highWater \wedge \neg methane) \Rightarrow \Diamond pumpOn)$ |
| #27 | $\neg(\Diamond(highWater \wedge \neg methane))$ |
| #28 | $((\Box\Diamond readCommand) \wedge (\Box\Diamond readAlarm) \wedge (\Box\Diamond readLevel)) \Rightarrow (\Box((highWater \wedge \neg methane) \Rightarrow \Diamond pumpOn))$ |
| #29 | $\Box((highWater \wedge \neg methane) \Rightarrow \neg\Diamond pumpOn)$ |
| #30 | $\neg\Diamond\Box(\neg pumpOn \wedge highWater)$ |
| #31 | $((\Box\Diamond readCommand) \wedge (\Box\Diamond readAlarm) \wedge (\Box\Diamond readLevel)) \Rightarrow (\neg\Diamond\Box(\neg pumpOn \wedge highWater))$ |
| #32 | $\neg\Diamond\Box(\neg pumpOn \wedge \neg methane \wedge highWater)$ |
| #33 | $((\Box\Diamond readCommand) \wedge (\Box\Diamond readAlarm) \wedge (\Box\Diamond readLevel)) \Rightarrow (\neg\Diamond\Box(\neg pumpOn \wedge \neg methane \wedge highWater))$ |
| #34 | $\Box((pumpOn \wedge highWater \wedge \Diamond lowWater) \Rightarrow (pumpOn U lowWater))$ |
| #35 | $\neg\Diamond(pumpOn \wedge highWater \wedge \Diamond lowWater)$ |
| #36 | $\Box(lowWater \Rightarrow (\Diamond\neg pumpOn))$ |
| #37 | $((\Box\Diamond readCommand) \wedge (\Box\Diamond readAlarm) \wedge (\Box\Diamond readLevel)) \Rightarrow (\Box(lowWater \Rightarrow (\Diamond\neg pumpOn)))$ |
| #38 | $\neg\Diamond\Box(pumpOn \wedge lowWater)$ |
| #39 | $((\Box\Diamond readCommand) \wedge (\Box\Diamond readAlarm) \wedge (\Box\Diamond readLevel)) \Rightarrow (\neg\Box\Diamond(pumpOn \wedge lowWater))$ |
| #40 | $\Box((\neg pumpOn \wedge lowWater \wedge \Diamond highWater) \Rightarrow ((\neg pumpOn) U highWater))$ |
| #41 | $\neg\Diamond(\neg pumpOn \wedge lowWater \wedge \Diamond highWater)$ |
| **Elevator** (58,945,690 FTS states, 256 valid variants) | |
| #1 | $\neg\Box\Diamond progress$ |
| #2 | $\neg\Box\Diamond f0 \vee \neg\Box\Diamond f1 \vee \neg\Box\Diamond f2 \vee \neg\Box\Diamond f3$ |
| #3 | $\neg\Box\Diamond p0at0 \vee \neg\Box\Diamond p0at1 \vee \neg\Box\Diamond p0at2 \vee \neg\Box\Diamond p0at3$ |
| #4 | $\Box(fb2 \Rightarrow (\Diamond f2))$ |
| #5 | $\Box\Diamond progress \Rightarrow (\Box(fb2 \Rightarrow (\Diamond f2)))$ |
| #6 | $\Box\Diamond progress \Rightarrow (\Box(fb2 \Rightarrow (\Diamond(f2 \wedge dopen))))$ |
| #7 | $\Box\Diamond progress \Rightarrow (\neg\Diamond\Box f2)$ |
| #8 | $\Box\Diamond(progress \vee waiting) \Rightarrow (\neg\Diamond\Box f2)$ |
| #9 | $\Box\Diamond(progress \vee waiting) \Rightarrow (\neg\Diamond\Box f0)$ |
| #10 | $\neg\Diamond((cb0 \vee cb1 \vee cb2 \vee cb3) \wedge \neg(p0in \vee p1in) \wedge dclosed)$ |
| #11 | $\Box\Diamond progress \Rightarrow (\neg\Diamond\Box dclosed)$ |
| #12 | $\Box\Diamond progress \Rightarrow (\neg\Diamond\Box(p0to3 \wedge dclosed))$ |
| #13 | $\Box\Diamond progress \Rightarrow (\neg\Diamond\Box dopen)$ |
| #14 | $\Box\Diamond(progress \vee waiting) \Rightarrow (\neg\Diamond\Box dopen)$ |
| #15 | $((\Box\Diamond(progress \vee waiting)) \wedge (\Box\Diamond(fb0 \vee fb1 \vee fb2 \vee fb3))) \Rightarrow (\neg\Diamond\Box dopen)$ |
| #16 | $\neg\Diamond(p0in \wedge p1in \wedge dclosed)$ |
| #17 | $\neg\Diamond\Box(p0in \wedge dclosed)$ |
| #18 | $\Box\Diamond progress \Rightarrow (\neg\Diamond\Box(p0in \wedge dclosed))$ |
| **CFDP** (1,801,581 FTS states, 56 valid variants) | |
| #1 | $\Diamond fileReceived$ |
| #2 | $(\Diamond eofReceived) \Rightarrow \Diamond fileReceived$ |
| #3 | $((\Diamond eofReceived) \wedge (\Diamond nakReceived)) \Rightarrow \Diamond fileReceived$ |
| #4 | $((\Diamond eofReceived) \wedge (\Box\Diamond nakReceived)) \Rightarrow \Diamond fileReceived$ |
| #5 | $\Box(finSend \Rightarrow fileReceived)$ |

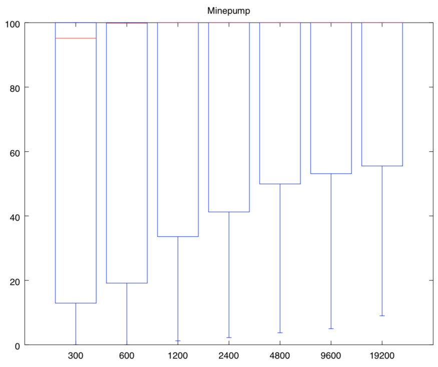

(a) Minepump (family-based SMC)

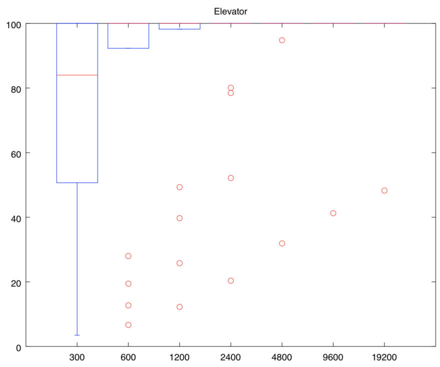

(b) Elevator (family-based SMC)

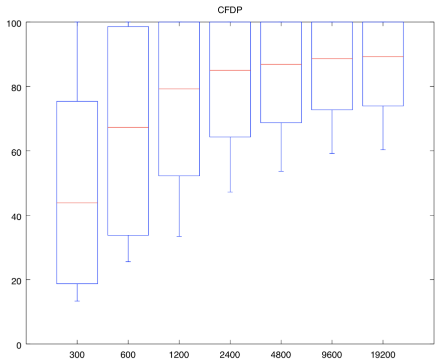

(c) CFDP (family-based SMC)

Fig. 2: Detection rate of the buggy variants achieved by our SMC method, in the three case studies and using different sample sizes. In each figure, the x-axis is the number of samples.

## 5 Results

### 5.1 RQ1: Detection Rate

Figure 2 shows as boxplots, for each case study and over all checked properties, the percentage of buggy variants for which family-based SMC found a counterexample. We provide those boxplots for different number $M$ of samples.

In the case of Minepump and Elevator, the median detection percentage is 100% starting from $M = 1200$ and $M = 600$, respectively. Further increasing the number of samples raises the 0.25 percentile. In Minepump and for $M = 1200$, there are 18/41 properties for which SMC could not detect all buggy variants. Increasing $M$ improves significantly the percentage of buggy variants detected by $SMC$ for all these properties, although there remain undetected variants in 15 of them even with $M = 19,200$. This illustrates that our assumption regarding

$p_{min}$ was inappropriate for those properties: counterexamples are rarer than we imagined. The elevator study yields even better results: at $M = 600$, SMC detects all buggy variants for 10/18 properties; this number becomes 14/18 at $M = 2,400$ and 17/18 at $M = 9,600$. As for the remaining property, SMC with $M = 19,200$ detects 50% of the variants on average and we observe that this percentage consistently increases as we increase $M$.

The results for CFDP are mixed: while the median percentage goes beyond 80% as soon as $M = 1,200$, it tends to saturate when increasing the number of samples. The 0.25 percentile still increases but also seems to reach an asymptotic behaviour in the trials with the highest $M$. A detailed look at the results reveals that for $M \geq 1,200$, SMC cannot identify all buggy variants for only two properties: #3 (9 buggy variants) and #4 (4 buggy variants). At $M = 19,200$, SMC detects 5.43 and 3.14 buggy variants for those two properties, respectively. Further doubling $M$ raises these numbers to 6.36 and 3.26. This indicates that the non-detected variants have few counterexamples, which are rare due to the tinier state space of those variants. The computation resources required by SMC to find such rare counterexamples with high confidence are higher than model-checking the undetected variants thoroughly. An alternative would be to direct SMC towards rare executions, leaning on techniques such as [10, 21].

> SMC can detect all buggy variants for 41 properties out of 59. For the remaining properties, however, SMC was unable to find the rare counterexamples of some buggy variants. This calls for new dedicated heuristics to sample those rare executions.

#### 5.2 RQ2: Efficiency

Next, we check how much execution time SMC can spare compared to the exhaustive search. Results are shown in Table 2. Overall, we see that SMC holds the potential to greatly accelerate the discovery of all buggy variants, achieving a total speedup of 526%, 1891% and 356% for Minepump, Elevator and CFDP, respectively. For more than half of the properties, the smallest number of samples we tried (i.e. 300) was sufficient for a thorough detection. Those properties are actually satisfied by all variants. The fact that SMC requires such a small number of samples means that the same bug lies in all the variants (as opposed to each variant violating the property in its own way). On the contrary, Minepump property #31 is also violated by all variants but requires a much higher sample number, which illustrates the presence of variant-specific bugs.

Interestingly, the benefits of SMC are higher in the Elevator case (the largest of the three models), achieving speedups of up to 16,575%. A likely explanation is that the execution paths of the Elevator model share many similarities, which means that a single bug can lead to multiple failed executions. By sampling randomly, SMC avoids exploring thoroughly a part of the state space that contains no bug and, instead, increases the likelihood to move to interesting

Table 2: Least numbers of samples (in our experiments) that allowed detecting all buggy variants and corresponding execution time. `Full` refers to an exhaustive search of the search space. Only properties that are violated by at least one variant and for which SMC found all buggy variants are shown.

| | | **SMC** | | **Full** | | |
|---|---|---|---|---|---|---|
| **Property** | **# Samples** | **# States** | **Time** | **# States** | **Time** | **Speedup** |
| Minepump #1 | 600 | 25332 | 0.18 | 92469 | 1.33 | 739% |
| Minepump #2 | 300 | 12553 | 0.10 | 24908 | 1.06 | 1060% |
| Minepump #4 | 300 | 2383 | 0.03 | 103933 | 3.10 | 10333% |
| Minepump #5 | 1200 | 48714 | 0.32 | 76040 | 1.03 | 322% |
| Minepump #7 | 300 | 2469 | 0.03 | 18482 | 0.21 | 700% |
| Minepump #8 | 300 | 2757 | 0.03 | 4646 | 0.05 | 167% |
| Minepump #9 | 300 | 2758 | 0.03 | 8263 | 0.08 | 267% |
| Minepump #10 | 600 | 15191 | 0.11 | 55936 | 0.58 | 527% |
| Minepump #12 | 300 | 2356 | 0.03 | 811 | 0.02 | 67% |
| Minepump #14 | 300 | 2915 | 0.04 | 989 | 0.02 | 50% |
| Minepump #15 | 300 | 2389 | 0.03 | 2673 | 0.05 | 167% |
| Minepump #16 | 300 | 4102 | 0.04 | 1917 | 0.03 | 75% |
| Minepump #18 | 300 | 2604 | 0.03 | 125 | 0.01 | 33% |
| Minepump #19 | 600 | 25027 | 0.18 | 143540 | 2.69 | 1494% |
| Minepump #20 | 300 | 3864 | 0.03 | 40 | 0.01 | 33% |
| Minepump #25 | 2400 | 67620 | 0.50 | 346935 | 6.12 | 1224% |
| Minepump #26 | 300 | 2708 | 0.03 | 4382 | 0.05 | 167% |
| Minepump #27 | 300 | 2450 | 0.03 | 3702 | 0.04 | 133% |
| Minepump #28 | 2400 | 58382 | 0.43 | 99780 | 1.28 | 298% |
| Minepump #30 | 300 | 300 | 0.03 | 3648 | 0.05 | 167% |
| Minepump #31 | 9600 | 165802 | 1.29 | 61185 | 1.03 | 80% |
| Minepump #32 | 300 | 2684 | 0.03 | 4110 | 0.05 | 167% |
| Minepump #41 | 300 | 5732 | 0.05 | 3886 | 0.04 | 80% |
| **Total** | | **461092** | **3.60** | **1062400** | **18.93** | **526%** |
| Elevator #1 | 300 | 4371 | 0.03 | 105883 | 0.52 | 1733% |
| Elevator #2 | 600 | 226813 | 1.14 | 437252 | 2.48 | 218% |
| Elevator #3 | 4800 | 1736781 | 7.67 | 14822853 | 103.22 | 1346% |
| Elevator #4 | 300 | 4403 | 0.04 | 1194568 | 6.63 | 16575% |
| Elevator #5 | 300 | 7719 | 0.05 | 1305428 | 7.76 | 15520% |
| Elevator #6 | 300 | 7061 | 0.05 | 1202204 | 6.89 | 13780% |
| Elevator #7 | 600 | 25021 | 0.12 | 732684 | 4.33 | 3608% |
| Elevator #8 | 600 | 26120 | 0.13 | 204934 | 1.19 | 915% |
| Elevator #9 | 300 | 3142 | 0.03 | 39086 | 0.28 | 933% |
| Elevator #11 | 300 | 3278 | 0.03 | 91 | 0.02 | 67% |
| Elevator #12 | 9600 | 1502419 | 6.53 | 1954924 | 11.12 | 170% |
| Elevator #14 | 2400 | 141753 | 0.61 | 7889584 | 52.88 | 8669% |
| Elevator #15 | 2400 | 142405 | 0.69 | 7889753 | 57.64 | 8354% |
| Elevator #16 | 2400 | 955206 | 4.02 | 28551923 | 182.25 | 4534% |
| Elevator #17 | 1200 | 100755 | 0.38 | 516230 | 3.53 | 929% |
| Elevator #18 | 4800 | 510145 | 1.94 | 486694 | 3.00 | 155% |
| **Total** | | **5397392** | **23.46** | **67334091** | **443.74** | **1891%** |
| CFDP #1 | 300 | 50206 | 0.20 | 87937 | 1.71 | 855% |
| CFDP #2 | 1200 | 117897 | 0.52 | 102842 | 0.85 | 163% |
| **Total** | | **168103** | **0.72** | **190779.00** | **2.56** | **356%** |

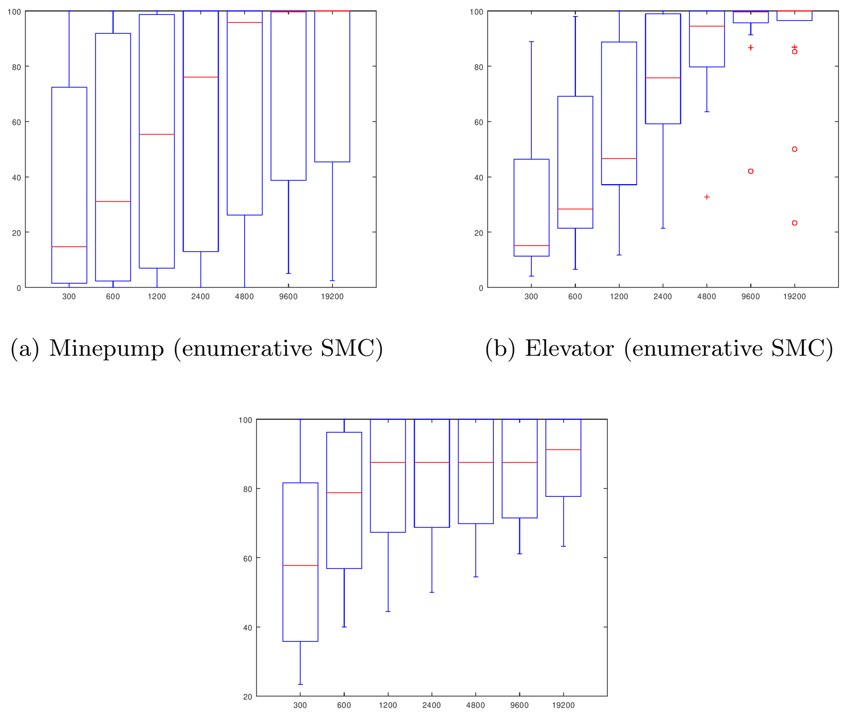

(a) Minepump (enumerative SMC)

(b) Elevator (enumerative SMC)

(c) CFDP (enumerative SMC)

Fig. 3: Detection rate of the buggy variants achieved by classical SMC applied variant by variant, in the three case studies and using different sample sizes. In each figure, the x-axis is the number of samples.

(likely-buggy) parts. A striking example is property #16 (satisfied by half of the variants), where SMC reduces the verification time from 3 minutes to 4 seconds.

> Where SMC can detect all buggy variants, it can do so with more efficiency compared to exhaustive search, for 33/41 properties, achieving speedups of multiple orders of magnitude.

#### 5.3 RQ3: Family-based SMC versus Enumerative SMC

Figure 3 shows the detection rate achieved the enumerative SMC for the three case studies and different numbers of samples, while the results of the family-based SMC were shown in Figure 2. In the Minepump and Elevator cases, enumerative SMC achieves a lower detection rate than family-based SMC. In both cases, a Student t-test with $\alpha = 0.05$ rejects, with statistical significance, the hypothesis that the two SMC methods yield no difference in error rate. One can observe, for instance, that, with 600 samples, enumerative SMC achieves a median detection rate of 31.13%, while family-based SMC achieved 99.86%. This tends to validate our hypothesis that family-based SMC is more effective as the variants share more executions. Indeed, on average, one state of the Minepump is shared by 3.55 variants.

In the case of CFDP, however, enumerative SMC performs systematically better (up to 13.95% more). Still, the difference in median detection rate tends to disappear as more executions are sampled. Nevertheless, CFDP illustrates the main drawback of family-based SMC: it can overlook counterexamples in variants with fewer behaviours. In such cases, enumerative SMC might complement family-based SMC by sampling from the state space of specific variants.

> Family-based SMC can detect significantly more buggy variants than enumerative SMC, especially when few lassos are sampled. Yet, enumerative SMC remains useful for variants that have a tiny state space compared to the others and can, thus, complement the family-based method.

### 6 Conclusion

We proposed a new simulation-based approach for finding bugs in VIS. It applies statistical model checking to FTS, an extension of transition systems designed to model concisely multiple VIS variants. Given an LTL formula, our method results in either collecting counterexamples for multiple variants at once or proving the absence of bugs. The algorithm always converges, up to some confidence error which we quantify on the FTS structure by relying on results for the coupon collector problem. After implementing the approach within a state-of-the-art tool, we study empirically its benefits and drawbacks. It turns out that a small number of samples is often sufficient to detect all variants, outperforming an exhaustive search by an order of magnitude. On the downside, we were unable to find counterexamples for some faulty variants and properties. This calls for future research, exploiting techniques to guide the simulation towards rare bugs/events [7,10,21] or towards uncovered variants relying, e.g., on distance-based sampling [22] or light-weight scheduling sampling [19]. Nevertheless, the positive outcome of our study is to show that SMC can act as a low-cost-high-reward alternative to exhaustive verification, which can provide thorough results in a majority of cases.
